# MicroRNA MultiTool: A Software for Identifying Modified and Unmodified Human microRNA Using Mass Spectrometry

**DOI:** 10.3390/ncrna3010013

**Published:** 2017-03-16

**Authors:** Zhonghao Cui, Norman H. L. Chiu, Dickson M. Wambua

**Affiliations:** 1 Department of Chemistry and Biochemistry, University of North Carolina at Greensboro, Greensboro, NC 27402, USA; zhjcui@gmail.com (Z.C.); prof.chiu@gmail.com (N.H.L.C.); 2 Joint School of Nanoscience and Nanoengineering, University of North Carolina at Greensboro, Greensboro, NC 27402, USA

**Keywords:** microRNA, MicroRNA MultiTool, modification, RNA modification, mass spectrometry

## Abstract

microRNA (miRNA) are short endogenous non-coding RNA that play a crucial role in post-transcriptional gene regulation and have been implicated in the initiation and progression of 160+ human diseases. Excellent analytical methods have been developed for the measurement of miRNA by mass spectrometry. However, interpretation of mass spectrometric data has been an incapacitating bottleneck in miRNA identification. This study details the development of MicroRNA MultiTool, a software for the identification of miRNA from mass spectrometric data. The software includes capabilities such as miRNA search and mass calculator, modified miRNA mass calculator, and miRNA fragment search. MicroRNA MultiTool bridges the gap between experimental data and identification of miRNA by providing a rapid means of mass spectrometric data interpretation.

## 1. Introduction

microRNA (miRNA) are a class of short endogenous non-coding RNA 16–28 nucleotides in length that play a crucial role in post-transcriptional gene regulation by either triggering degradation or preventing the translation of targeted messenger RNAs. With the recent discovery that miRNA can control over 60% of human gene expression, it is not surprising that the field of miRNA biology has quickly gained considerable interest within the last decade [1]. miRNA can regulate a variety of cellular processes [2,3], and have been implicated in the initiation and progression of 160+ human diseases [4]. For this reason, miRNAs have been intensely studied for their potential use as disease biomarkers or therapeutic targets [5].

Numerous analytical methods have been developed for the identification and characterization of miRNAs. These methods include polymerase chain reaction, Northern blots, microarrays, and mass spectrometry (MS), among others. MS has been an important tool for the analysis of nucleic acids due to its accuracy, sensitivity, efficiency, and ability to perform high throughput measurements. Furthermore, the ability of MS to measure the intrinsic property of miRNA (i.e., its molecular mass) gives it an added advantage in miRNA measurements over the alternative molecular biology techniques that rely on the measurement of secondary signals. The availability of high throughput assays for the analysis of miRNA has made MS a more appealing technique for miRNA measurements. We recently demonstrated that MS can be used for the accurate detection and de novo sequencing of miRNA [6]. Nakayama et al. also reported a method for the direct identification of miRNA using high resolution mass spectrometry in conjunction with database searching [7]. Matrix-assisted laser desorption/ionization (MALDI) results in the generation of dominantly singly charged ions that are relatively easy to interpret, and is therefore the preferred choice for oligonucleotide analysis [8].

There are currently more than 2500 human miRNA reported in the literature [9]. Several miRNAs can coexist within the same sample, and therefore, parallel analysis of multiple miRNAs is unavoidable. The mass spectrometric data generated from miRNA measurements can therefore be complex, and manual data processing is tedious and challenging [10]. Recent studies have also revealed that just like other forms of RNA, miRNA can undergo nucleotide modification which plays a key role in miRNA maturation and functions [11]. miRNA nucleotide modifications are important, since modified miRNA are known to target transcripts that are different than those targeted by unmodified miRNA [12]. According to MODOMICS (a comprehensive database resource for RNA modifications), there are more than 100 distinct nucleotide modifications [13]. Whereas most of the current molecular biology techniques are unable to distinguish miRNA modifications, MS has been used for the characterization of different RNA post-transcriptional modifications, including identification of type and exact position of the RNA modification [14,15]. The phenomenon of miRNA modification adds a layer of complexity to the miRNA data interpretation, further underlining the importance of developing analytical methods and appropriate software capable of identifying both modified and unmodified miRNA [16].

With these challenges in mind, the spectral interpretation of mass spectrometric data can quickly turn out to be the bottleneck in miRNA analysis [9]. The success of MS as a tool for the analysis of miRNA will therefore strongly depend on the development of relevant software with the ability to properly interpret and analyze the large-scale, highly-dimensional data that is generated from MS measurements [17].

To address the challenges associated with MS data analysis of both post-transcriptionally modified and unmodified miRNA, we present here the development of a user-interactive software, termed as MicroRNA MultiTool (available in the Supplementary Materials) written using Java programming language. MicroRNA MultiTool has a graphical user interface that allows the user to directly utilize mass spectral data in order to identify miRNA by sequence, name, mass, accession number, and/or RNA modification if any. Since the molecular mass of miRNA can be directly used for its identification, MicroRNA MultiTool can potentially eliminate the need of performing tandem mass spectrometry on a miRNA for the sole purpose of its identification.

## 2. MicroRNA MultiTool Functionalities

**Figure 1:**
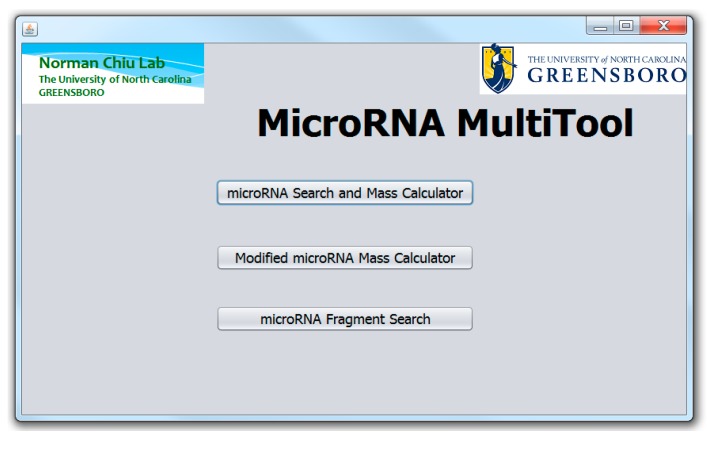
MicroRNA MultiTool graphical user interface.

The human miRNA sequences were obtained from miRBase, a database which serves as a primary repository for published miRNAs [1,18], while RNA modifications were obtained from MODOMICS, a comprehensive database for RNA modification [19]. As shown in Figure 1, the MicroRNA MultiTool graphical user interface has three primarily features (a) miRNA search and mass calculator; (b) modified miRNA mass calculator, and (c) miRNA fragment search. Help buttons are strategically placed with appropriate information to guide the user on the functions offered by the program.

### 2.1. miRNA Search and Mass Calculator

**Figure 2:**
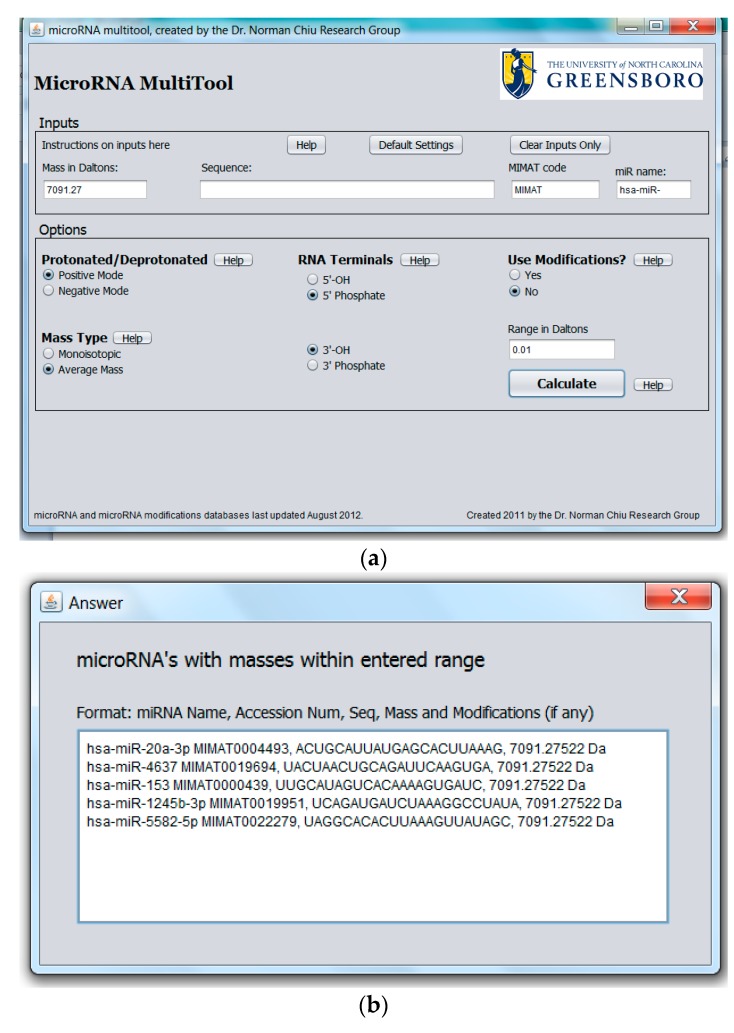
(**a**) Graphical user interface for microRNA (miRNA) search and mass calculator function showing a mass input in the “Mass in Daltons” field; (**b**) Output results for miRNA search and mass calculator function showing the result obtained by entering a mass of 7091.27 in the “Mass in Dalton” field in Figure 2a.

The purpose of the miRNA search and mass calculator function is to match the data obtained from a mass spectrometric measurement to all known human miRNA. This feature is especially important when miRNA are to be identified from mass spectral data with no other information available. The graphical user interface for miRNA search and mass calculator and its output are shown in Figure 2a,b, respectively. In the interface shown in Figure 2a, the “Mass in Daltons” field accepts numerical values and is provided for searching modified or unmodified miRNA. To search for the identity of a miRNA, a specific mass obtained from mass spectrometric data is entered into the “Mass in Daltons” field. In the “Options” panel, the ionization mode used to acquire the MS data (i.e., “Protonated” for positive ion mode or “Deprotonated” for negative ion mode) is selected. A choice is also provided between a search for monoisotopic or average mass as well as the type of 3′ and 5′ terminal groups on the miRNA. In the “Options” panel, the option of “Use Modification” is available for searching modified miRNA, with the default set at “No” under “Use Modifications” for searching unmodified miRNA. Clicking on “Calculate” returns the result of the analysis. The result of the search is a list of all unmodified miRNAs matching the entered mass together with their names, accession numbers, sequence, and molecular masses, as shown in Figure 2b.

**Figure 3:**
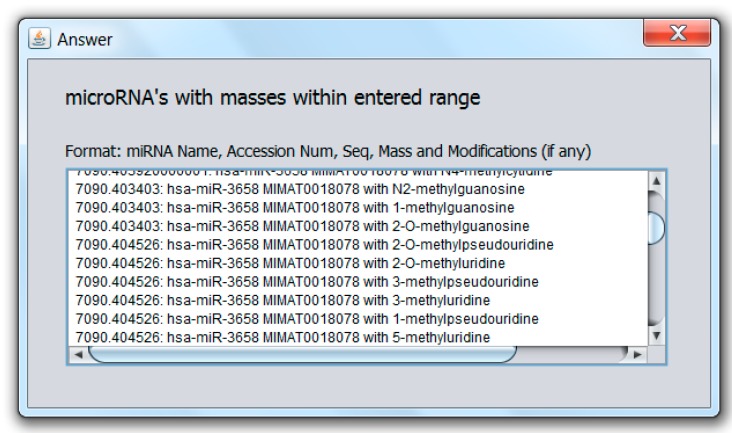
Modified miRNA search results output.

A unique function of the miRNA search and mass calculator compared to other RNA mass calculators is the ability to identify miRNA that may carry a modification on one of the nucleotides as well as the possible modification. This is done by first entering the mass as obtained from the mass spectrometric measurement in the “Mass in Daltons” field shown in Figure 2a, selecting desired options in the “Options” panel as explained above, followed by selecting “Yes” under “Use Modifications”, then “Calculate”. The software algorithm then scans each miRNA in the database and evaluates the sum of the miRNA mass plus the mass of any one of the known miRNA modifications. This is done for all known human miRNA and RNA modifications. The output consists of the modified miRNA mass, name, accession number, and modification, as shown in Figure 3.

Depending on the type of mass spectrometer used, the mass accuracy of the measured value can vary greatly. In order to account for different levels of mass accuracy, the user is offered the option to define a ± range in Daltons (Da) around the measured mass, which the program algorithm can use to search for matching miRNA masses. The defaults in the options panel are set at positive ionization mode, average mass, 5′ phosphate, 3′ hydroxyl, no modification, and ±0.01 Da range. After all options have been made, the user clicks the “Calculate” button to obtain the results.

**Figure 4:**
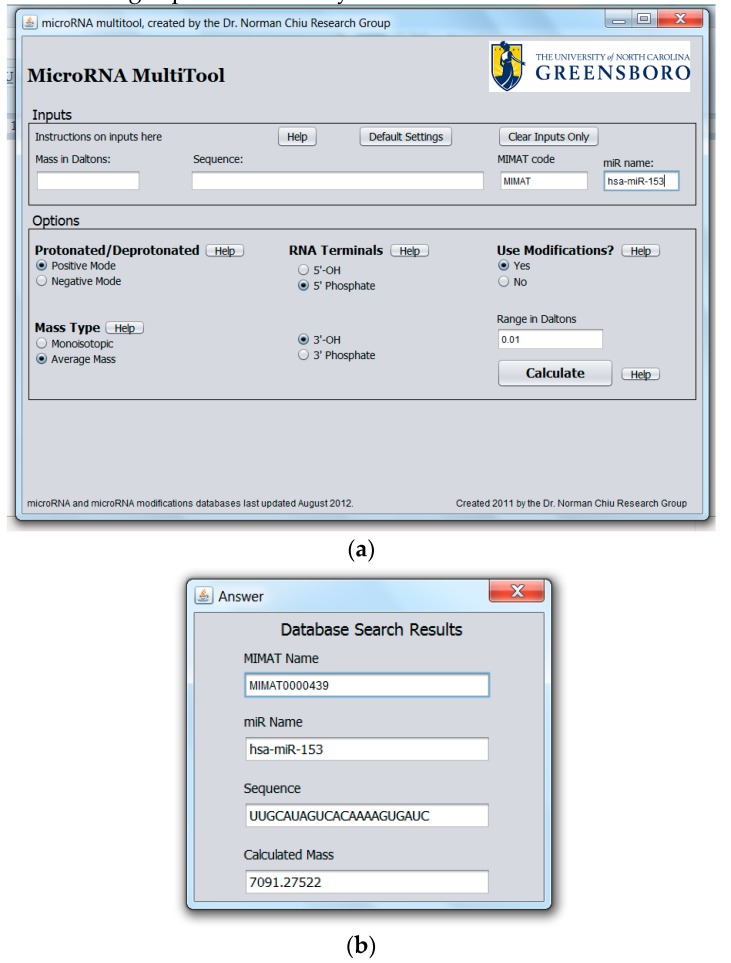
(**a**) MicroRNA MultiTool search and mass calculator user interface showing the various fields for data entry, with an entry of hsa-miR-153 in the “miR name” field; (**b**) MicroRNA MultiTool search and mass calculator output of hsa-miR-153 as entered in the “miR name” field in Figure 4a.

The developed MicroRNA MultiTool software can also be used to retrieve or confirm the relevant information on each available human microRNA. The “Sequence”, “MIMAT”, and “miR Name” fields of the miRNA search and mass calculator can accept RNA sequence (A,G,C,U), accession number, and miRNA name entries, respectively. If the entry matches any known miRNA, the program returns all parameters associated with the miRNA, including its accession number, name, sequence, and mass. The choices on the “Options” panel can be selected as previously explained. Spaces entered between inputs are automatically ignored. An example with hsa-miR-153 entered in the name field is shown in Figure 4a, while the output is shown in Figure 4b.

### 2.2. Modified miRNA Mass Calculator

**Figure 5:**
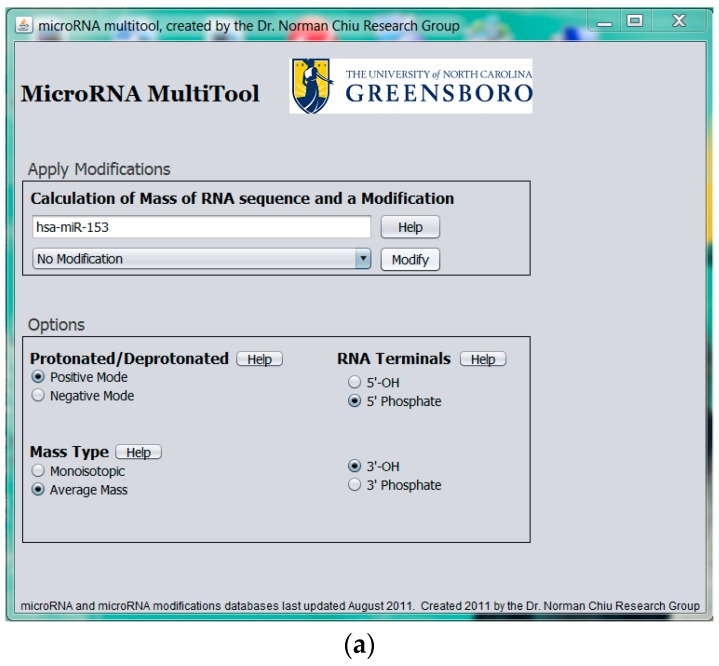

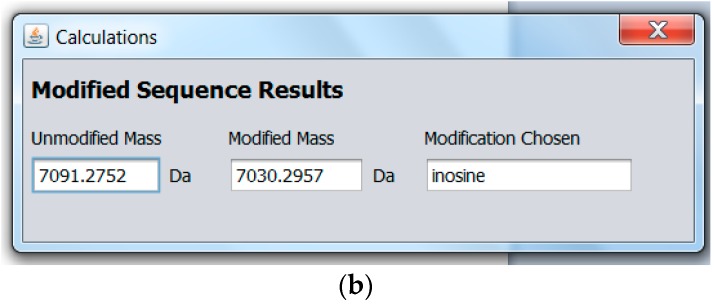
(**a**) MicroRNA MultiTool modified miRNA mass calculator user interface showing unmodified entry of hsa-miR-153; (**b**) MicroRNA MultiTool modified miRNA mass calculator output showing the unmodified mass of hsa-miR-153, a modification, and the resulting mass.

To provide pre-experimental miRNA sample analysis information, MicroRNA MultiTool offers a novel feature whose purpose is to simulate expected mass spectrometric data of modified miRNA. As shown in Figure 5a, the data entry field provided in the Modified miRNA mass calculator interface accepts input in the form of miRNA name (e.g., hsa-miR-153). A drop-down menu with RNA modifications is provided, from which the user can select a single modification. The program calculates the total mass of the miRNA and the modification and returns an output as shown in Figure 5b, consisting of the mass of the unmodified miRNA selected, mass of modified miRNA, and the modification chosen. The biological sample can then be monitored for the output mass from MicroRNA MultiTool during experimental analysis.

### 2.3. miRNA Fragment Search

**Figure 6:**
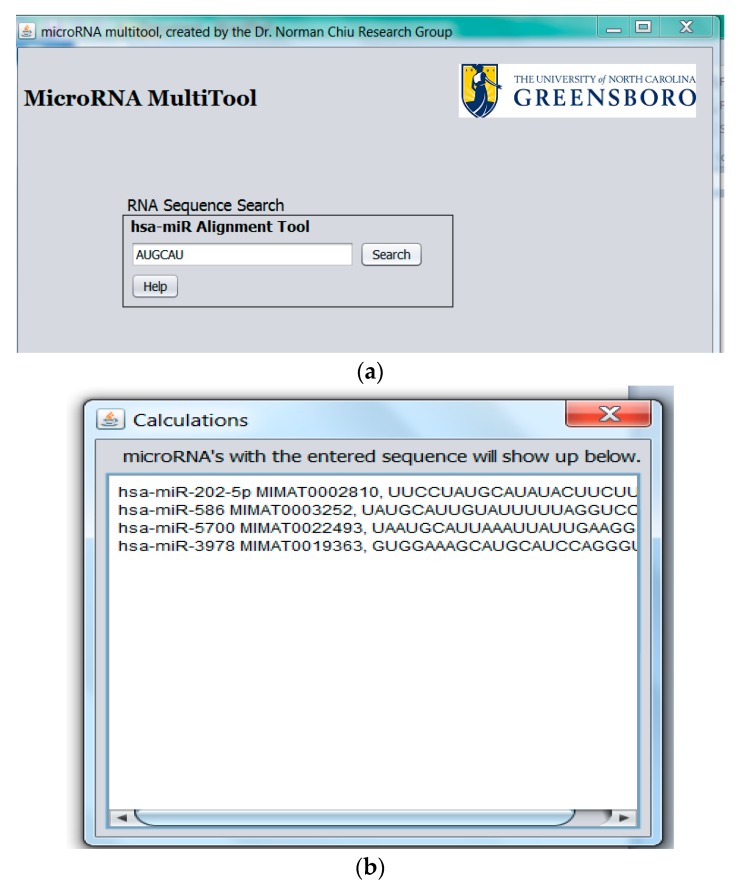
(**a**) miRNA fragment search feature; (**b**) miRNA fragment search feature output.

The miRNA fragment search feature shown in Figure 6a allows the alignment of an RNA fragment against all known human miRNA. Once miRNA containing the queried sequence are identified, MicroRNA MultiTool returns the matching miRNA name, accession number, and sequence(s) of all miRNAs containing the input sequence fragment as shown in Figure 6b.

## 3. Discussion

The current strategy for identifying miRNA by MS relies on first obtaining the mass of miRNA, followed by tandem mass spectrometry (MS/MS) in which the miRNA is fragmented through different ways (e.g., collision-induced dissociation, CID). The resulting fragment ions are then used to deduce a sequence that can be matched to possible miRNA identity [20]. Even though a few groups have recently reported the complete sequence assignment of RNA greater than 19 nucleotides long by MS/MS, this process does not always yield sufficient sequence coverage to enable miRNA identification [21,22,23]. The determination of miRNA identity from MS data rather than MS/MS is therefore a simpler, straightforward, and more appealing approach to this challenge. Furthermore, the bioinformatics tools for the direct identification of miRNA from MS data are lacking. MicroRNA MultiTool bridges the gap and provides a way of identifying miRNA without carrying out the MS/MS measurements.

Currently, there are several bioinformatics tools available for assisting in the interpretation of RNA MS data. McCloskey’s group developed the Mongo Oligo Mass Calculator, a multipurpose program for the calculation of oligonucleotide molecular masses, CID fragments, and endonuclease and exonuclease RNA digestion fragments [24]. Simple Oligonucleotide Sequencer (SOS) from the same group is a useful tool for the determination of oligonucleotide sequences from CID MS/MS data [25]. The Compas program developed by Oberacher et al. uses comparative sequencing of oligonucleotides for the sequence verification of nucleic acids by matching the predicted CID-MS/MS data with the multiply-charged anionic peaks obtained from an analyzed sample [26]. These programs help interpret RNA MS data. Ariadne is a software tool that was developed by Nakayama et al., and is geared towards the identification of RNA sequences from tandem mass spectrometric data of RNase digests. It has been shown to work with transfer RNA with and without post-transcriptional modification [27]. However, none of these programs is capable of identifying miRNA from MS data. MicroRNA MultiTool offers a simple yet practical solution to this challenge by allowing users to match masses obtained in an MS experiment against molecular masses of all known human miRNA in order to identify the measured miRNA. This approach of direct identification of miRNA from mass spectral data eliminates the need to perform MS/MS experiments for the sole purpose of miRNA identification.

Due to the short nature of miRNAs, isobaric miRNAs (i.e., miRNAs with different RNA sequences but identical molecular masses) do exist. We recently showed that 55% of all human miRNA are structural isomers [28]. Under these circumstances, MicroRNA Multitool can be used to narrow down the identity of isobaric miRNA by returning a list of all the isobaric miRNA with the queried mass. For the absolute identification of isobaric miRNAs, currently existing methods of isolation and identification can be used. We have previously demonstrated that isobaric miRNA can be identified by capturing them using complementary DNA probes followed by MS measurement [29]. To help in the identification of miRNA when a partial sequence is available, the MicroRNA MultiTool sequence alignment tool can be used. The algorithm searches for miRNA bearing queried partial sequence and matches it to known miRNA. Partial sequences of miR-153 that were obtained through mass spectrometric measurements in our laboratory [6] were queried in MicroRNA Multitool, and the results of matching number of miRNA are tabulated in Table 1. From these results, it is clear that hsa-miR-153 (whose sequence is 5′ pUUGCAUAGUCACAAAAGUGAUC-OH 3′) can be positively identified using the unique fragment [5′ pUUGCAUAGUC 3′], which is 45.4% of the miRNA sequence. MicroRNA MultiTool therefore provides the means for incomplete sequencing data to be used for miRNA identification.

The presence of post-transcriptionally modified nucleotides in various forms of RNA such as transfer RNA (tRNA) has been known for decades. So far, there are more than 100 known RNA modifications [19]. The specific roles of most modifications still remain unknown; however, modifications are well conserved across evolution, and are thought to be important for the general wellness of the organisms. Similar to the other forms of RNA, miRNA are susceptible to different forms of post-transcriptional enzymatic editing and covalent modifications [30]. Nucleotide modifications are capable of altering the way in which miRNA interact with target mRNA molecules [31]. This can result in changes in gene regulation [32] and the development of various diseases (e.g., cancer) [33]. Even though most of the RNA modifications are functionally and structurally important, the conventional and currently available molecular biology-based techniques such as microarrays, Quantitative Reverse-Transcriptase Polymerase Chain Reaction (qRT-PCR), and Northern blots or sequencing methods are incapable of detecting or identifying different types of RNA modifications [34]. Except for the “mass silent” pseudouridylation modification which involves the isomerization of uridine to pseudouridine without any molecular mass changes [35], most miRNA modifications result in mass shifts with respect to the unmodified miRNA. Since the mass shifts are characteristic of the modification, MS analysis is a useful tool for the identification of modified miRNA as well as the specific modification involved [36]. For the identification of modified miRNA, the MicroRNA MultiTool algorithm uses a database of all known human miRNA together with known RNA modifications and computes all possible permutations for modified miRNA within the user queried mass. The output includes modified miRNA mass, name, accession number, and modification involved. To accurately predict a modified miRNA, the program does not require the sequence, name, or accession number of the miRNA. The only input required is the mass of the modified miRNA as obtained from MS experiments. The mass is used to automatically generate a list of all modified miRNA together with the modification(s) involved. Usually, spectra generated from MALDI-MS are easier to analyze because they are mainly dominated by singly charged ions [37]. This is also true when using MicroRNA MultiTool; however, in other cases where multiply charged ions are observed (such as in electrospray ionization MS), the spectra should be deconvoluted to the singly charged ions. Based on one of our recent publications, isomeric human miRNA which have identical molecular mass may exist in the same biological sample [28]. When mass spectrometry is used to analyze such biological sample, tandem mass spectrometric measurement can be used to differentiate and identify specific isomeric miRNA. The data analysis will therefore require the use of additional software that would support the tandem mass spectrometric data. It is important to note that there are many available online programs for assisting the analysis of tandem mass spectrometric data, which is why such a feature has not been incorporated in the MicroRNA MultiTool.

## 4. Conclusions

This study aimed at simplifying the identification of miRNA from mass spectrometric data. MicroRNA MultiTool provides the ability to search the identity of miRNA using a single parameter (e.g., miRNA mass, sequence, accession number, or name) one at a time and return the other three related parameters. Given that miRNA therapeutic agents are already being explored as possible therapies for numerous disease conditions, the rapid identification of miRNA will play a key role in the advancement of such therapies. The development of computational tools for the interpretation of MS data can greatly reduce the complexity associated with analysis of MS data while increasing the speed with which the data can be processed.

## Figures and Tables

**Table 1:** Alignment of hsa-miR-153 using MicroRNA MultiTool.

5′ pUUGCAUAGUCACAAAAGUGAUC-OH 3′ (hsa-miR-153)		
Partial Sequence	# of miRNAs with Partial Sequence	% Sequence Coverage
pUUGCAUAGUC	1	45.4
pUUGCAUAGU	3	40.9
pUUGCAUAG	3	36.3
pUUGCAUA	5	31.8
pUUGCAU	17	27.2
pUUGCA	58	22.7
pUUGC	174	18.1
pUUG	597	13.6
pUU	1101	9.0
pU	1722	4.5

## Supplementary Materials

MicroRNA MultiTool software developed in this study is available in the supplementary materials at www.mdpi.com/2311-553X/3/1/13/s1.
